# The Role of Single Nucleotide Polymorphisms at the Arg399Gln Locus of the *XRCC1* Gene in Patients with Non-Small Cell Lung Cancer (NSCLC)

**DOI:** 10.3390/ijms26136540

**Published:** 2025-07-07

**Authors:** Beata Smolarz, Bartosz Cieślik-Wolski, Józef Kozak, Honorata Łukasiewicz, Dariusz Samulak, Dariusz Trzmielak, Hanna Romanowicz, Marianna Makowska

**Affiliations:** 1 Laboratory of Cancer Genetics, Department of Pathology, Polish Mother’s Memorial Hospital Research Institute, 93-338 Lodz, Poland; hanna-romanowicz@wp.pl; 2 Department of Thoracic Surgery, Medical University of Lodz, Memorial Copernicus Hospital, 93-338 Lodz, Poland; bartoszcw@gmail.com (B.C.-W.); szpital@kopernik.lodz.pl (J.K.); 3 Department of Nursing, Faculty of Medicine and Health Sciences, The President Stanisław Wojciechowski Calisia University, 62-800 Kalisz, Poland; honorata.lukasiewicz@wp.pl; 4 Department of Obstetrics and Gynecology and Gynecological Oncology, Regional Hospital in Kalisz, 62-800 Kalisz, Poland; samulakd@wp.pl; 5 Department of Obstetrics, The President Stanisław Wojciechowski Calisia University, 62-800 Kalisz, Poland; 6 Department of Science, Polish Mother’s Memorial Hospital Research Institute, 93-338 Lodz, Poland; dariusz.trzmielak@iczmp.edu.pl; 7 Faculty of Management, University of Lodz, 90-237 Lodz, Poland; 8 Department of Anesthesiology and Operative Intensive Care Medicine, Charité-Universitätsmedizin Berlin, Corporate Member of Freie Universität Berlin, Humboldt-Universität zu Berlin, 10117 Berlin, Germany; marianna.makowska@yahoo.com

**Keywords:** non-small cell lung cancer, *XRCC1*, polymorphism

## Abstract

In recent years, an increasingly important role in the etiopathogenesis of lung cancer has been attributed to genetic predisposition. Current genetic research suggests that the increased risk of this cancer may be due to gene polymorphism within repair genes. In the case of lung cancer, observations about genes involved in the DNA repair system by cutting bases of nitrogen—base excision repair (BER)—seem to be interesting. Most attention has been devoted to the *XRCC1* gene, which coordinates the various stages of BER. The aim of this study was to assess the role of the single nucleotide polymorphism Arg399Gln in the *XRCC1* gene as a factor influencing the risk of lung cancer. The study involved 118 patients with non-small cell lung cancer (NSCLC). The control group consisted of 60 people who did not have cancer. The study proved that the polymorphism of the *XRCC1* gene is characterized by a statistically significant relationship with the onset of cancer. There were no statistically significant differences between the Arg399Gln polymorphism of the *XRCC1* gene and risk factors for non-small cell lung cancer, such as age, sex, smoking and its duration, or place of residence, as well as between the histological type of the tumor or its severity. Detailed analysis of three genotypes—Arg/Arg, Arg/Gln, and Gln/Gln—showed that the incidence of particular genotypes in the group of patients was, respectively, 16.10%, 27.12%, and 58.78%. In the case of the Gln/Gln genotype, the most common associated histopathological type was squamous cell carcinoma, and in the case of adenocarcinoma, the most common genotype was Arg/Arg. It was estimated that each Arg allele reduced the chance of tumor occurrence to 0.48 times the reference value, i.e., the Gln/Gln genotype class for the Arg/Gln genotype and the Arg/Gln genotype for the Arg/Arg genotype. The relationship between the male sex and the occurrence of cancer remained insignificant, in contrast to the presence of nicotinism. Studies suggest that the Arg399Gln polymorphism of the *XRCC1* gene has limited prognostic significance in non-small cell lung cancer.

## 1. Introduction

Epithelial lung cancers have been divided into two main morphological types: small cell lung carcinoma (SCLC) (10–15% of cases) and non-small cell lung carcinoma (NSCLC) (85–90% of all lung cancers). SCLC is more aggressive and spreads faster. NSCLC is less aggressive, with a higher chance of cure, especially if detected at an early stage [1]. SCLC is usually treated with chemotherapy and radiation, while NSCLC, in addition to these methods, can also be treated with surgery [2]. The prognosis in SCLC is usually worse than in NSCLC, especially in the advanced stages. However, in the case of NSCLC, appropriate treatment, especially surgical treatment at an early stage, may give a good prognosis [3]. NSCLC is not histopathologically homogeneous. The most common subtypes of NSCLC are adenocarcinoma (about 40% of lung cancers), squamous cell carcinoma (about 30%), and large cell carcinoma (5–10% of cases). The literature emphasizes that in about 20–25% of patients there is a mixed weave within the tumor [4]. In addition, in some cases, due to the ambiguous histopathological picture, the cancer subtype cannot be determined. In such cases, only on the basis of the expression of immunohistochemical markers is it possible to diagnose NSCLC corresponding to adenocarcinoma or NSCLC corresponding to squamous cell carcinoma, and in the remaining cases the final diagnosis is defined as unspecified cancer (NOS—not otherwise specified) [5].

SCLC occurs almost exclusively in smokers. SCLC tends to spread rapidly to other organs such as lymph nodes, bones, brain, liver, and adrenal glands [6].

Nicotinism is the cause of lung cancer in about 15% of smokers [7,8]. The reason why not every smoker develops malignant lung cancer is the role of genetic predisposition in the etiopathogenesis of cancer. Previous genetic studies have drawn attention to the importance of genomic polymorphism in relation to the incidence of the disease, the course of the disease, or the results of cancer treatment [9].

Polymorphisms can be divided into single nucleotide polymorphisms (SNPs) and mini- and microsatellite sequence polymorphisms [10]. It should be emphasized that many years of research on various types of cancer have shown that the occurrence of SNPs can lead to both earlier onset of proliferative disease, its altered response to treatment, as well as more frequent familial occurrence of a given cancer. Polymorphisms can also occur within genes that metabolize xenobiotics, genes of repair systems, and genes involved in important processes for the cell, i.e., proliferation, growth, differentiation, or the ability to undergo apoptosis [11,12].

The major biochemical pathway to clear oxidative damage to DNA is the DNA Repair System by Cutting Out Nitrogenous Bases—base excision repair (BER) [13].

Polymorphic DNA repair genes are mostly classified as low penetrance genes. This means that the product of a single gene usually has a slight effect on the response to the xenobiotic, but the accumulation of altered alleles may have a major impact on this response [14,15].

Therefore, the most important research is on the variability of many DNA repair genes and their combinations. Research on the interaction between DNA repair genes and protein-coding genes involved in xenobiotic detoxification also seems to be important. In the future, common knowledge about genetic polymorphism may enable mass screening tests to be carried out, which would serve as an indicator of susceptibility to occupational exposure or long-term nicotinism in patients predisposed to lung cancer [16,17]. There are many analyses in the literature on gene polymorphism and the associated probability of developing lung cancer [18,19]. Much attention is paid to the *XRCC1* gene, whose protein product, recruited by DNA glycosidase enzymes, coordinates individual stages of one of the DNA repair systems—BER [20,21].

The *XRCC1* gene is located on the longer arm of chromosome 19 at the 19q13.2 locus, occupies about 31.9 kb, and contains 17 exons, encoding a 633-amino acid protein. In various tissues of the body, this gene is expressed at a high level [22]. Several polymorphisms have been identified in the *XRCC1* gene that may play an important role in the development of non-small cell lung cancer [23,24,25]. So far, about 40 polymorphic sites have been found within the *XRCC1* gene. Fourteen of them cause a change in the encoded amino acid, and four occur with a frequency of more than 3%. The main polymorphisms are *XRCC1* Arg194Trp, *XRCC1* Trp194Trp, *XRCC1* Arg280His, and *XRCC1* Arg399Gln [26,27,28]. These polymorphisms were analyzed in terms of the overall risk of lung cancer and smoking, without taking into account other risk factors or treatment methods. Data on the above-mentioned polymorphisms are ambiguous and variable among different populations.

An extensive analysis conducted in six Eastern European countries showed that the *XRCC1* Arg194Trp genotype is associated with a small risk of lung cancer [29]. The study, which included 109 patients diagnosed with lung cancer in the Chinese population, indicated an increased risk of lung cancer for the *XRCC1* Trp194Trpgenotype [30]. The Arg399Gln polymorphism in the *XRCC1* gene is associated with the incidence of lung cancer in North Indians. Screening for this polymorphism can help target predisposed people to prevent cancer [31].

*XRCC1*’s involvement in the BER pathway is critical for repairing DNA damage caused by platinum agents. *XRCC1* polymorphisms correlate with altered repair capacity, affecting clinical outcomes and resistance to platinum-based chemotherapy in patients with NSCLC [26]. The use of machine learning approaches showed promising results in predicting lung cancer based on *XRCC1* polymorphisms. In the study, polymorphism data of five single nucleotide polymorphisms of the DNA repair gene *XRCC1* (*XRCC1* 399, *XRCC1* 194, *XRCC1* 206, *XRCC1* 632, *XRCC1* 280) from the population of northern India, along with data on smoker status, were considered as inputs to the proposed ensemble model to predict the risk of individual susceptibility to lung cancer. Results indicate the potential of the proposed team model to predict cancer risk based on *XRCC1* SNP data [23].

Lung cancer, as a disease with a diverse etiology, requires a thorough analysis at the molecular level—genetic, epigenetic, and functional. Currently, there is no doubt that one of the main causes of lung cancer is a decrease in the activity of DNA repair systems, mainly BER, but a detailed analysis of the participation of individual elements of this system in the pathogenesis of this disease is needed. A thorough understanding of these factors may contribute to the development of sensitive tests necessary for effective diagnosis and prevention of this type of cancer. Being able to establish an accurate genetic profile of a patient with non-small cell lung cancer could allow for targeted, personalized therapy for patients with this severe disease [32,33,34,35].

The aim of this study is to determine the prognostic significance of the single nucleotide polymorphism Arg399Gln of the *XRCC1* gene in patients with non-small cell lung cancer.

## 2. Results

Most of the patients were chronic smokers who underwent anatomical lung resection, i.e., lobectomy. In postoperative histopathological examination, the most commonly diagnosed cancer was adenocarcinoma. The most frequently observed stage of cancer was stage I, and the most common grade of malignancy was stage G2. The groups differed statistically significantly in the frequency of smoking. The control group was statistically significantly younger than the study group and was characterized by a statistically insignificantly higher percentage of women. The epidemiological characteristics of the study and control groups are presented in Table 1, and the clinical-pathomorphological characteristics are presented in Table 2.

The frequency of the *XRCC1* gene genotype was analyzed. In the control group, genotype frequencies were consistent with the distribution expected according to the Hardy–Weinberg equilibrium (*p* = 0.3889), which means that the control group was collected in an adequate, random manner, and the genotype frequencies observed in healthy subjects could be a reference point for comparison with the group of cancer patients. The results are summarized in Table 3.

Statistically significant differences in genotype frequencies were found between the compared groups.

According to the above data, it was concluded that carriage of the Arg allele is a protective factor against the onset of cancer, because the odds ratio for carriers of the Arg allele (Arg/Arg homozygotes and heterozygotes) was 0.67 (95%CI 0.36–1.24). However, this effect was not statistically significant (*p* = 0.2020), most likely due to the size of the study group.

A comparison of the histopathological type of tumors observed in patients differing in the genotype of the *XRCC1* gene polymorphism is presented in Table 4.

Genotype frequencies did not differ significantly between histological types of the tumor (*p* = 0.6487). In the comparison of carriers of the Arg variant vs. Gln/Gln homozygotes, significance was also not observed (*p* = 0.3809).

Genotype frequencies did not differ significantly between the clinical stages of the disease (*p* = 0.9949). In comparison, for carriers of the Arg variant vs. Gln/Gln homozygotes, significance was also not observed (*p* = 0.9875). A comparison of the clinical stages according to genotype is presented in Table 5.

Genotype frequencies did not differ statistically significantly between the degree of histopathological malignancy (*p* = 0.7868). In comparison, for carriers of the Arg variant vs. Gln/Gln homozygotes, significance was also not observed (*p* = 0.7137). The frequency of histological malignancy of the tumor depending on the genotype of the polymorphism studied is presented in Table 6.

Genotype frequencies did not exhibit a statistically significant difference depending on the number of pack-years (*p* = 0.6757). In comparison, for carriers of the Arg variant vs. Gln/Gln homozygotes, significance was also not observed (*p* = 0.6026) (Table 7).

A multivariate analysis using logistic regression was performed to assess the strength of the association of the genotype of the *XRCC1* gene polymorphism studied, taking into account the confounding effects of age, male sex, and nicotinism. The results are presented in Table 8.

The relationship between the male sex and the occurrence of cancer remained insignificant, in contrast to the presence of nicotinism.

## 3. Discussion

Lung cancer is currently the most commonly diagnosed malignant neoplasm and is the main cause of mortality in people with cancer both in Poland and worldwide (approx. 1.8 million cases and approx. 1.6 million deaths annually worldwide) [36].

One of the most common types of lung cancer is non-small cell lung cancer. It is responsible for the majority of cancer-related deaths worldwide. There has been a significant improvement in the treatment of NSCLC in the last ten years. Innovative systemic treatment options and new surgical and radiotherapeutic techniques were implemented. Systematic screening of at-risk populations using low-dose computed tomography (CT) scans has been introduced. In locally advanced NSCLC, the combination of different perioperative strategies and adjuvant immunotherapy increases cure rates. In the case of metastases, the implementation of new drugs can prolong the control of the disease while maintaining quality of life [37]. A special unit in advanced NSCLC is the oligometastatic NSCLC (omNSCLC). NSCLC in the oligometastatic stage means that the cancer has spread to several places in the body. For oncogene-independent omNSCLC, systemic treatment should be considered, reserving local consolidative treatment (LAT) for non-progressive patients. In the case of oncogene-dependent omNSCLC, the LAT must be carefully evaluated to determine whether to reserve it for oligoprogression or to apply it in advance. It also depends on the type of genomic alterations, the location of the lesions, and the patients’ symptoms. When it comes to LAT techniques, surgery and stereotactic body radiotherapy (SBRT) remain the most effective and safest options [38].

The development of predictive clinical and genetic markers is necessary for the advancement of individualized treatment concepts [37].

Small Cell Lung Cancer (SCLC) can be classified into molecular subtypes based on the expression of key transcription factors, leading to the identification of SCLC-A (high expression of the transcription factor ASCL1), SCLC-N (high expression of NEUROD1), SCLC-Y (high expression of the transcription factor YAP1), and SCLC-P (high expression of POU2F3) subtypes [39]. SCLC is a heterogeneous disease, and understanding the different subtypes and their interactions is important. Monitoring tumor evolution and treatment response using molecular biology techniques may be essential for personalized therapy.

For NSCLC, novel molecular agents are identified as promising therapeutic targets, including KRAS non-G12C, RAF/MEK, HER3, Nectin-4, folic acid receptor alpha, ITGB6, and PRMT5. New targets for targeted therapy have been proposed, such as NSD3, ATR, FGFR, NRF2, and AXL. Evaluation of new strategies, including targeted and immunological therapeutic approaches, is ongoing and will form the basis for future treatment strategies [40].

For patients with resectable NSCLC in the absence of EGFR mutations or ALK gene rearrangements, perioperative immunotherapy-based treatment is now considered the standard [41]. Three-year adjuvant treatment with osimertinib improves disease-free survival (DFS) and overall survival (OS) and reduces central nervous system (CNS) recurrence in patients with EGFR-mutated non-small cell lung cancer resection at stage IB–IIIA. The use of osimertinib after chemoradiotherapy in patients with stage III EGFR-mutated non-small cell lung cancer showed a significant improvement in progression free survival (PFS). In the case of ALK-positive, 2-year treatment with alectinib has been shown to clearly improve DFS compared to standard adjuvant chemotherapy in patients with stage IB (≥4 cm)–IIIA non-small cell lung cancer resection. Studies are being conducted to determine the optimal duration of adjuvant TKI therapy as well as neoadjuvant TKI strategies for EGFR- and ALK-positive disease and (neo)adjuvant targeted therapy in patients with actionable genetic changes other than EGFR or ALK [41].

Currently, lung cancer is almost three times more common in men, but increased trends in the incidence of the female population in recent years have led to a decrease in this proportion, especially in countries with a high human development index [42]. Such a low survival rate in lung cancers is associated with a long, asymptomatic period of the disease and the initiation of diagnostics at a significant stage of its advancement. Of the 67 countries that participated in the CONCORD-3 project, the 5-year survival rate for lung cancer ranged from 2.2% in Libya, 16.5% in Switzerland, 18.7% in the United States, and 30.1% in Japan [43].

These alarming statistics, especially concerning the female population, prompt, on the one hand, more focused attention on the implementation of population-based screening programs, and, on the other hand, the search for new molecular biomarkers [44].

The introduction of screening has always been aimed at screening high-risk populations to detect precancerous conditions or diseases in the asymptomatic phase. The use of traditional imaging radiology methods in screening studies, used, for example, in the randomized NLST study, was the basis for the development of European recommendations [45].

Previous attempts to use modern biomarkers of neoplastic transformation, such as *K-RAS* or *TP53* gene mutations, telomerase activity, or microsatellite instability, in screening tests have not been the basis for introducing tests into screening programs [46].

On the one hand, the above molecular disorders contribute to a personalized understanding of the carcinogenesis process, but the insufficient sensitivity and specificity of molecular tests cannot constitute the “gold standard” of lung cancer screening. The clinical value of the above tests performed alone or in combination with classical imaging radiology methods requires further research.

It is still necessary to very precisely determine the population at high risk of developing lung cancer, and for this it seems necessary to combine imaging radiology methods with a panel of molecular biomarkers. Therefore, it is justified to search for new molecular markers in order to be able to detect low-stage tumors to an even greater extent and apply radical treatment to them, giving the best prognosis [47].

The study analyzed the importance of single nucleotide polymorphisms of the Arg399Gln gene of the *XRCC1* gene in patients with non-small cell lung cancer.

On the one hand, SNP determines the occurrence of DNA variability but very often contributes to the formation of such changes in the DNA sequence that contribute to the occurrence of many diseases, including malignant tumors [48].

In this study, the frequency of occurrence of polymorphic variants of *XRCC1* genes in patients with NSCLC and in the control group was analyzed, and the results obtained were correlated with epidemiological, clinical, and pathomorphological features that could affect the incidence of NSCLC in Polish patients.

Of the dozens of possible polymorphisms of the *XRCC1* gene in question, almost half are responsible for changing the protein encoded by this gene. The main polymorphisms are *XRCC1* Arg194Trp, *XRCC1* Try194Trp, *XRCC1* Arg280His, and *XRCC1* Arg399Gln [26].

In the scientific literature, these polymorphisms have been analyzed in terms of the overall risk of lung cancer and smoking, without taking into account other risk factors or treatment methods [23,49,50,51].

The basis for the choice of topic was the fact that lung cancer is very often asymptomatic, which confirms that classic methods of detecting this cancer should be enriched with molecular assay panels.

When planning the study, a set of various clinical, epidemiological, and pathomorphological features were selected in order to obtain a uniform picture of the significance of the above polymorphism. A detailed analysis was carried out by determining the correlation between the polymorphism of the *XRCC1* Arg399Gln gene and age, sex, nicotinism, place of residence, and type of cancer or its stage. All this in order to determine a specific utility algorithm for determining the gene polymorphism mentioned above.

It should be emphasized that such an indication of mutually correlating features in the studied patient population may be used to perform a situational analysis of a specific patient in the future. In the age of personalized medicine, such an analysis involving the study of genetic polymorphisms and their relationship with the risk of non-small cell lung cancer would be crucial to making the right therapeutic decisions. In addition, the indication of the genetic profile that may increase the risk of developing lung cancer could allow researchers to distinguish a population with a particularly high risk of developing the disease. In this group, it would be justified to systematically perform tests aimed at detecting the tumor at an early, asymptomatic stage, guaranteeing full curability. Such a selected population of patients could be covered by a special screening program, e.g., using spiral computed tomography.

Such a procedure, modeled on NSLC and NELSON tests, would allow for early detection of cancer in a particularly predisposed population, analyzing not only the number of burnt pack-years [52,53]. In the Internal Early Lung Cancer Action Program(I-ELCAN) study, the estimated 10-year survival rate for people diagnosed with stage I lung cancer was 88%, and 92% for resection [54].

In this study, it was confirmed that smoking increased the risk of cancer, which has been proven in studies on both Polish and international populations. Smoking tobacco became popular in the twentieth century, and a significant link between long-term smoking and the occurrence of cancer was demonstrated about 70 years ago. In Poland, smoking is the cause of 93% of lung cancer cases in men and about 76% of cases in women over 35 years of age [55].

Results from a prospective study involving two one-million-strong samples of U.S. citizens showed that the relative risk of lung cancer among smokers compared to nonsmokers was 24 for men and 12.5 for women [56].

Smoking and older age in standard clinical situations are correlated. The longer people smoke, the greater their chance of developing lung cancer. Each cigarette smoked is a source of reactive oxygen and nitrogen species. The above compounds are produced continuously both by the cells of our body and by those introduced through carcinogenesis. In a properly functioning body, the produced reactive oxygen and nitrogen species are neutralized by well-functioning antioxidant systems [57].

In the event of imbalance—homeostasis—or the occurrence of chronic oxidative stress, reactive oxygen and nitrogen species have a damaging effect on cells and tissues by inducing damage in the DNA/RNA chain. As long as the repair systems are efficient, damage is eliminated, for example, by widely described systems: BER (base excision repair), NER (nucleotide excision repair), or TLS (translesion synthesis) [58].

Smoking, especially in elderly patients, means that not all molecular damage is correctly recognized by the above-mentioned repair systems. When the resulting changes exceed the “corrective” abilities as a result of too many damages or malfunctioning proteins of the repair systems due to, among other things, mutations in the coding genes, the following mutations arise, inducing a complicated and multi-stage process of carcinogenesis. Literature data confirm that the first studies on the occurrence of polymorphisms were dedicated to determining the future effectiveness of systemic therapies using drugs such as cisplatin [59].

During chemotherapy, a cancer cell develops mechanisms for acquiring drug resistance. The study of gene polymorphisms can help predict the occurrence of molecular mechanisms in the entire pathway of the emergence of drug resistance, such as reduced uptake of cisplatin by target cells (blocking the function of hCTR1), inactivation of cisplatin by glutathione and enzymes involved in its transformation, disorders of repair systems (BER, NER, or MMR), and disorders of anti-apoptotic systems, e.g., BCL-2, TP53 [58].

Studies confirm that single polymorphic variants of genes involved in the NER repair system do not affect the prognosis in cancer patients [60].

The presence of at least several polymorphisms at the same time may correlate with the clinical course, but their impact on the results of chemotherapy is not determined. Therefore, the study of polymorphisms in the course of lung cancer is increasingly analyzed in terms of analyzing the risk of this disease. In this aspect, most literature reports concern the XRCC1 platform protein, which coordinates individual stages of the BER repair system [61,62].

It was shown that the change in the *XRCC1* 399Gln/Gln gene variant to *XRCC1* Arg399Gln was associated with an increased risk of lung cancer among Asians, but not among Caucasians. It also highlighted the fact that advances in the identification of new polymorphisms and the continued development of new genotyping techniques will facilitate the analysis of multiple genes on multiple DNA repair pathways in the future [31,63,64].

Studies of the *XRCC1* Gln399Gln polymorphism of the Polish population showed that the risk of death from lung malignancy was higher in adenocarcinoma patients with *XRCC1* Gln/Gln genotypes, although their frequency was low. The *XRCC1* allele 399Gln was also associated with a poor prognosis in stage II–IIIA and among the elderly [65].

It should be noted that the data for the polymorphisms mentioned above are ambiguous and variable among different populations.

Great hopes are associated with the analysis of many risk factors and the presence of SNPs as very valuable additional information to determine appropriate therapeutic management [66].

Therefore, in the presented paper, a panel of factors such as age, sex, tumor location, histopathological type, tumor stage, and tumor grade was taken into account for full analysis. The study showed that when all factors were taken together, only smoking and age increased the risk of cancer.

This confirms the statement that lung cancer occurs in only a small proportion of tobacco smokers, suggesting a genetic predisposition. On the other hand, the differing tendency to develop lung cancer may result from the presence of polymorphisms in genes responsible for, among others, the metabolism of carcinogens contained in nicotine smoke [67]. The study illustrated the fact that the presence of the Arg allele was characterized by a lower risk of developing the disease than in the control group.

Similar conclusions were observed in previous studies, which confirmed that the *XRCC1* Arg194Trp genotype in particular is associated with a low risk of lung cancer, while the Trp194Trp polymorphism of the *XRCC1* gene increases this risk [29,68,69].

The relationship between the male sex and the occurrence of cancer remained insignificant, in contrast to the presence of nicotinism. However, men constituted the majority of the cancer group according to current epidemiological data.

Since the beginning of the 1960s, mortality due to lung cancer among Polish women has quadrupled. On the one hand, this was the result of the spread of smoking, especially during and after World War II; on the other hand, some cohort studies indicate a greater susceptibility to carcinogens contained in tobacco smoke in women than in men, which may be related to hormonal factors [70]. In addition, there are differences in the proportion of histological forms of lung cancer between women and men. Adenocarcinomas are much more common in women, and squamous cell carcinomas in men [71]. Tobacco smoke has a damaging effect on the efficiency of the NER repair system, which is crucial for the formation of further damage in the complicated and multi-stage process of carcinogenesis [72]. The results of the study clearly confirm the effect of carcinogenic substances in cigarette smoke on the accelerated development of the disease compared to the control group.

In this study, a detailed analysis of the genotypes Arg/Arg, Arg/Gln, and Gln/Gln was performed. The frequency of occurrence of individual genotypes in the study group was 16.10%, 27.12%, and 58.78%, respectively. In the case of the Gln/Gln genotype, the most common associated histopathological type was SCC. In the case of AC, the most common genotype was Arg/Arg (>60%).

In addition, it was estimated that each Arg allele lowers the chance of cancer by 0.48 times the reference value, i.e., the Gln/Gln genotype for the Arg/Gln genotype and the Arg/Gln genotype for the Arg/Arg genotype.

The presence of the Arg allele, which was characterized by a lower risk of disease development than that of the control group, is also significant.

In addition, the multivariate analysis took into account the influence of genotypes on the TNM stage and malignancy grade, but no statistically significant correlations were observed.

The multivariate analysis using logistic regression showed that the effect of the Arg variant of the *XRCC1* gene polymorphism is strong enough to maintain its statistical significance in the multivariate analysis taking into account the effects of clinical confounding variables significantly associated with the occurrence of lung cancer, such as age, male sex, and nicotinism.

The results presented are encouraging, and it is advisable to continue further research. To confirm these findings, it is advisable to continue multicenter studies on large groups of patients with lung cancer. The creation of an algorithm or a so-called “clinical calculator” could provide the basis for the use of the above results in routine clinical practice.

Despite the change in the treatment strategy for NSCLC in recent years, lung cancer is still a cancer with a very poor prognosis. Therefore, correlating clinical, histopathological, and molecular factors with each other will be crucial to improve epidemiological indicators, especially in the female population. Despite the use of new drugs with mechanisms so different from classic chemotherapy, the best survival results are achieved by the use of thoracic surgery procedures in the early stages of cancer. Therefore, it is worth continuing the research so that it is useful in everyday clinical practice in the future.

## 4. Materials and Methods

The study included 118 patients with non-small-cell lung cancer operated on in 2016–2017 at the Clinical Department of Thoracic Surgery and Pulmonary Rehabilitation of the Nicolaus Copernicus Regional Multidisciplinary Oncology and Traumatology Center in Łódź.

**Figure 1:**
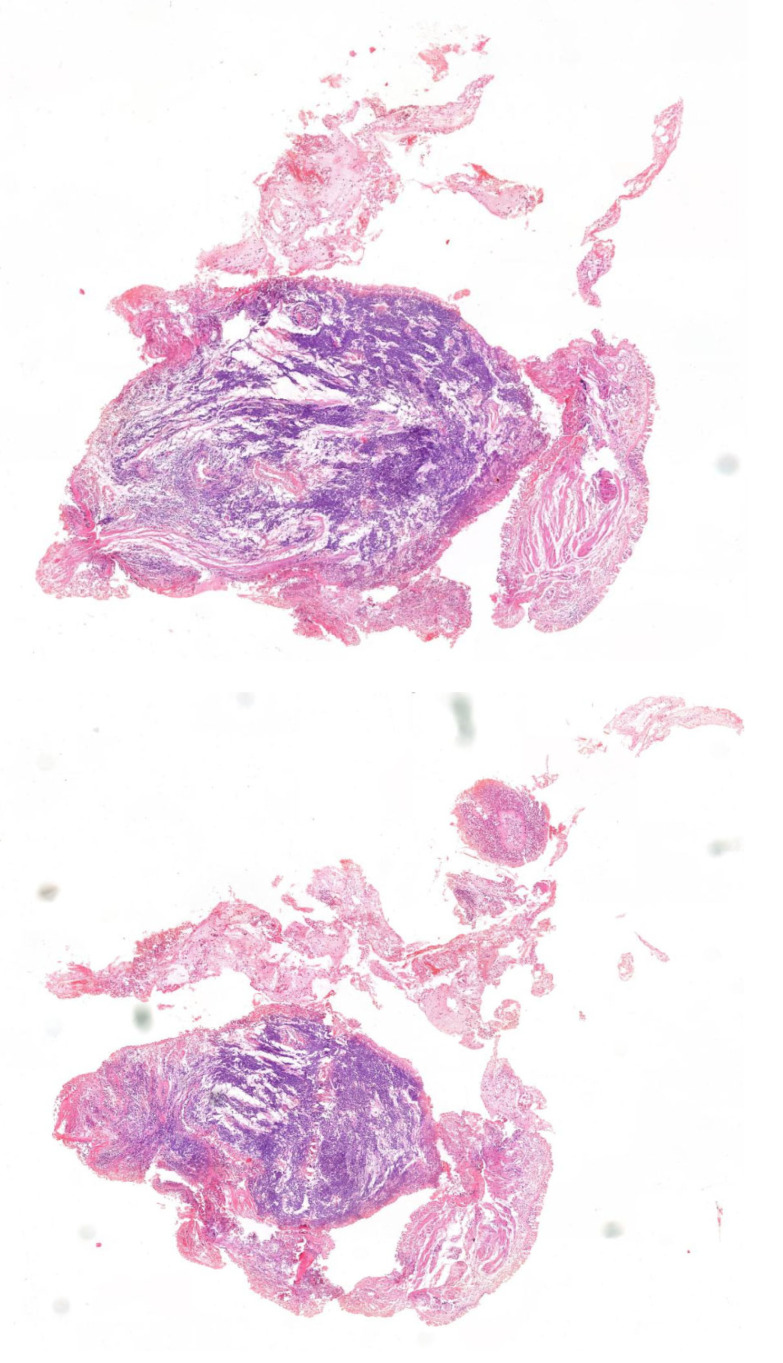
Lung cancer (hematoxylin–eosin staining) (from the Department of Pathology, Polish Mother’s Memorial Hospital Research Institute, Łódź, Poland). Image obtained from the scanner (Case Viewer 2.3, 3D Histech, Budapest, Hungary); magnification ×200.

Histopathological examination and classification of the tumor were carried out at the Department of PathomorphologySynevo in Łódź in accordance with the classification of the World Health Organization, while the stage of the tumor was carried out according to the eighth TNM scale in force since 2017, following UICC/AJCC criteria. The histological examinations were performed by experienced pathologists using a digital slide scanner (Sysmex, 3D Histech, Budapest, Hungary) and slide viewer software (Case Viewer 2.3, 3D Histech, Budapest, Hungary). Lung cancer sites were selected by a panoramic viewer (3D Histech) and used for testing (Figure 1). The study received an internal funding grant from the Operational Program Digital Poland MDB-MEDICAL DATA BANK (grant no. POPC.02.03.01-00-0091/19).

The control group consisted of 60 patients. The criterion for selection for this group was the exclusion of any cancer until the tests were conducted. All subjects gave written consent to participate in the study.

The study was approved by the Independent Bioethics Committee for Scientific Research at the Polish Mother’s Memorial Hospital Research Institute in Łódź (No. 362/2017).

The material for this study was DNA isolated from peripheral venous blood leukocytes. Blood samples were taken from patients before surgery and from control patients during routine health examinations. Blood was collected each time into vacuum tubes containing EDTA and stored at a temperature of −20 °C until DNA isolation was performed. The database of patients with NSCLC contained information collected on the basis of medical records, postoperative histopathological results and a questionnaire prepared for each patient. These included the patient’s age, sex, residence, nicotinism, type of surgery, type of cancer and its stage, metastatic lesions to lymph nodes, and genotype—Arg399Glnof the *XRCC1* gene polymorphism.

The control group database contained information such as gender, age, smoking data, place of residence, and the Arg399Gln *XRCC1* polymorphism.

### 4.1. Characteristics of Patients with NSCLC and Controls

The study group consisted of 118 patients with NSCLC. This group included 70 men (59%) and 48 women (41%). The mean age of the treated patients was 66 years (range 42 to 78 years).

In 109 patients (92.4%), information was obtained as to the place of residence in the last 10 years. In this group, 33 people (30.3%) lived in cities with a population of up to 10 thousand, 45 people (41.3%) lived in cities with a population between 10 and 100 thousand, while 31 people (28.4%) lived in agglomerations with more than 100 thousand inhabitants.

Information on cigarette smoking was also obtained. Only 9 people declared a complete lack of nicotinism. In the surveyed group, 109 answered affirmatively to the question asked in the survey about nicotinism. Sixty-nine people (63.3%) admitted to active smoking or quitting cigarettes up to 12 months before surgery. There were 40 former smokers, i.e., people who had quit smoking more than 12 months before the surgery (36.7%). Among people with nicotine addiction, it was possible to determine the approximate number of pack-years. Seventeen respondents reported that they had smoked less than 15 pack-years, 36 people declared the number of pack-years in the 15–30 range, while there were 56 chronic smokers who smoked more than 30 pack-years.

All patients qualified for surgery had previously undergone imaging tests (CT, PET/CT), bronchofiberoscopy, and spirometry. Preoperative diagnosis of lung malignancy was established in 87 patients (74%) by collecting material for histopathological examination by means of tweezer biopsy of endobronchial lesions, fine or core needle transthoracic biopsy, or transbronchial biopsy of the tumor/lymph nodes in EBUS-TBNA. In 19 (16%) patients, lung cancer was confirmed during intraoperative examination, and in 12 (10%) patients on the basis of the final histopathological examination after surgery.

The basic surgical procedure performed in patients from the study group was the removal of one lobe, i.e., lobectomy. It was performed in 97 patients (82%), and bilobectomy in 3 patients (3%). In 6 patients (5%), pneumonectomy was performed. A conservative procedure, i.e., segmentectomy or wedge resection, was performed in 12 patients (10%).

Among the operated patients, 64 cases (54.24%) were diagnosed with adenocarcinoma, 44 (37.29%) with squamous cell carcinoma, 4 (3.39%) with large cell carcinoma, and in 6 (5.08%) with mixed carcinoma.

In total, 4 patients were diagnosed with highly differentiated carcinoma (feature G1), 95 patients with moderately differentiated carcinoma (feature G2), and 19 patients with low-differentiated carcinoma (feature G3).

The stages of lung cancer according to the pTNM classification were as follows: IA1—9 patients; IA2—27; IA3—16; IB—14; IIA—9; IIB—32; IIIA—11.

On the basis of histopathological postoperative examinations, the T1a feature was found in 10 patients, T1b—30, T1c—19, T2a—22, T2b—12, T3—23, T4—2. The N0 feature was found in 94 patients, the N1 feature in 21, and the N2 feature in 3.

The control group consisted of 60 people who had not been diagnosed with cancer until the material for the test was collected. There were 28 men and 32 women in this group, and the average age was 55 years (range: 19–87 years). Twenty-three people from this group for the last ten years had lived in towns with fewer than 10 thousand inhabitants, 21 people in towns with 10–100 thousand inhabitants, and 13 in towns with more than 100 thousand inhabitants. In total, 39 people (65%) declared themselves smokers, and 19 people (35%) had never been active smokers.

### 4.2. DNA Isolation

DNA was isolated from the blood of subjects tested using the DNeasyBlood & Tissue Kit (Qiagen, Hilden, Germany) according to the test manufacturer’s instructions.

### 4.3. Genotyping

Based on the data available in the National Center for Biotechnology Information (http://www.ncbi.nlm.nih.gov/snp NCBI (SNP) 2 April 2019 database, the single nucleotide polymorphism Arg399Gln (rs25487) located in the *XRCC1* gene was selected. The polymerase chain reaction–restriction fragment length polymorphism (PCR-RFLP) method was used to study polymorphic variants of selected DNA repair genes. For the analysis of polymorphism, a reaction mixture with a volume of 50μLwas used, containing:

- 2 μL of genomic DNA;
- 5 μL of PCR bubble (TaKaRa, Kyoto, Japan);
- 4 μL dNTPs (10 mM)(TaKaRa, Kyoto, Japan);
- 1 U Taq polymerase (TaKaRa, Kyoto, Japan);
- 0.5 μL of appropriately selected primers (10 mM) (Polgen, Łódź, Poland);
- H_2_O (UltraClean PCR Water, MO BIO Laboratories, Carlsbad, CA, USA).

The amplification was performed in the Thermal Cycler PTC-100 TM (MJ Research, INC, Waltham, MA, USA) under appropriately selected conditions. Initial denaturation was carried out for 5 min at 95 °C, then a selected fragment of the studied gene was amplified during 35 cycles with the following parameters: 30 s at 95 °C, 30 s at 58 °C, and 30 s at 72 °C. Final elongation was carried out at 72 °C for 3 min. Table 9 shows the primer sequences and thermal conditions for PCR reactions.

The PCR product contained in a 10μLreaction mixture was incubated with 1 U of the restriction enzyme BcnI (NciI) (Thermo Scientific, Fermentas, Lithuania). The reactions were carried out according to the procedures recommended by the manufacturer: digestion temperature 37 °C for 16 h and enzyme inactivation temperature 65 °C for 20 min (Table 10).

### 4.4. Agarose Gel Electrophoresis

**Figure 2:**
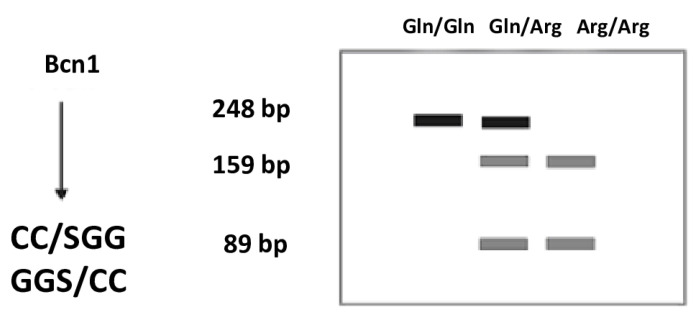
Diagram of the predicted sizes of DNA fragments obtained as a result of digestion with the restriction enzyme. Bp—base pairs.

**Figure 3:**
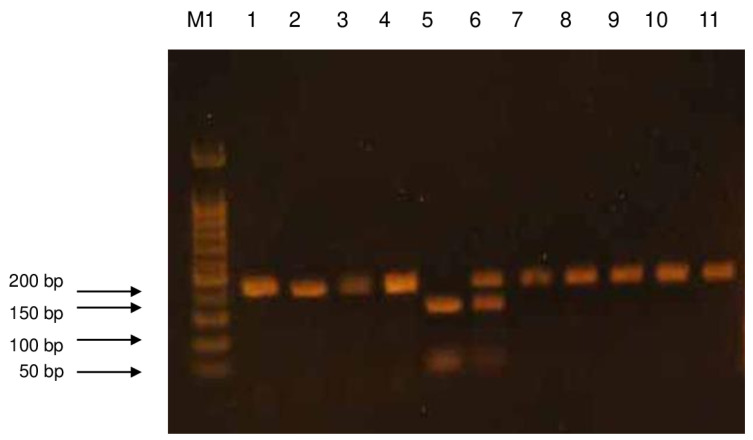
An example of electrophoretic separation of DNA fragments after digestion with the BcnI enzyme. Agarose gel separated products. M1—mass standard (50 bp DNA Ladder), 1–4, 7–11—patients homozygous Gln/Gln, 5—homozygous Arg/Arg patient, 6—heterozygous Arg/Gln patient.

The products of digestion with a restriction enzyme were separated using the electrophoresis technique in 2% agarose gel. The agarose used came from AppliChem (AppliChem GmbH, Darmstadt, Germany). DNA Ladder 50 bp from BIORON (Bioron GmbH, Ludwigshafen, Germany) was used as a mass standard. Electrophoretic separation was performed in a BIOMETRA apparatus at an electric field strength of 6 V/cm. The gel was stained with ethidium bromide (AppliChem GmbH, Darmstadt, Germany) at a concentration of 10 mg/mL in order to visualize the separated products, and then it was observed under UV light in the gel documentation apparatus (Syngen Biotech. Wroclaw, Poland). The obtained images were electronically saved as graphic files using the microDOC (Major science, Azure 200, Syngen, Wroclaw, Poland) system (Figure 2 and Figure 3). The Arg/Arg genotype corresponded to the 159.89 bp band, the Arg/Gln genotype to the 248, 159, and 89 bp bands, and the Gln/Gln genotype to the 248 bp band.

### 4.5. Methods of Statistical Analysis

The collected data were analyzed using the STATISTICA 13.1 statistical package licensed by the Medical University of Łódź. Continuous variables, due to the distribution significantly deviating from the normal (verified by the Shapiro–Wilk W test), are presented using medians and lower and upper quartile values (25–75%). Comparisons between the two groups were made using the Mann–Whitney U test. Comparisons between a larger number of groups were made using the Kruskall–Walis test with the post hoc Dunn–Bonferroni comparison procedure. Nominal variables are presented as observation counts and percentage values calculated in relation to the study and control groups. Comparisons were made using the chi-squared test. For low counts (<5 observations in any field of the bipartite table), an accurate, two-tailed Fisher test was performed to increase the conservativeness of the test. The frequency of genotypes in the control group was related to the distribution expected according to the Hardy–Weinberg law. If statistically significant (*p* < 0.05) or close to significant (*p* < 0.15) results were observed, the odds ratios for cancer were calculated.

In order to eliminate the confounding effect of clinical variables significantly different between the study and control groups, an analysis was performed using multivariate logistic regression taking into account genotype, age, sex, and smoking. The results of the model are presented as regression coefficients, odds ratios with 95% confidence intervals (95%CI), and *p*-values.

## 5. Conclusions

In this study, statistically significant differences in genotype frequencies were found between the compared groups of subjects and the control group. It was found that the incidence of the Arg allele or alleles is associated with a lower risk of non-small cell lung cancer, but no statistical significance was found in this respect. In addition, it was estimated that each Arg allele lowers the chance of cancer occurrence by 0.48 times the reference value, i.e., the Gln/Gln genotype class for the Arg/Gln genotype and the Arg/Gln genotype for the Arg/Arg genotype. Based on the conducted research, with the limitation of the low sample size, it can be assumed that the single nucleotide polymorphism Arg399Gln of the *XRCC1* gene has limited prognostic significance in non-small cell lung cancer and requires further studies.

## Figures and Tables

**Table 1:** Epidemiological characteristics of the study group of patients with lung cancer (n = 118) and the control group (n = 60) included in the study of the Arg399Gln polymorphism of the *XRCC1* gene.

Variable		Study Group (n = 118)		Control Group (n = 60)		*p*-Value
		Median	25–75%	Median	25–75%	
Age		66.5	62–70	60.5	41.5–69	0.0012
	Category	Number	%	Number	%	
**Sex**	woman	46	38.98%	32	53.33%	0.0681
	man	72	61.02%	28	47.67%	
**Smoking**	no	11	9.02%	19	33.93%	<0.0001
	yes	71	58.20%	37	66.07%	
	long quit	40	32.79%	0	0.00%	

**Table 2:** Clinical and pathological characteristics of the study group (n = 118) included in the Arg399Gln polymorphism study of the *XRCC1* gene.

Feature		Number	%
side	left	58	49.15
	right	60	50.85
type	glandular	64	54.24
	squamous cell	44	37.29
	mixed	6	5.08
	large cell	4	3.39
treatment	upper lobectomy	59	50.00
	lower lobectomy	33	27.97
	wedge resection	6	5.08
	middle lobectomy	5	4.24
	segmentectomy	6	5.08
	pneumonectomy	6	5.08
	lower bilobectomy	3	2.54
TNM	I	65	55.08
	II	42	35.59
	III	11	9.32
metastases to nodes	yes	24	21.05
	no	90	76.27
grading	G1	4	3.57
	G2	95	84.82
	G3	13	11.61

**Table 3:** Frequency of individual genotypes in the study group (n = 118) with lung and control cancers (n = 60) included in the Arg399Gln polymorphism study of the *XRCC1* gene.

	Patients		Control		
Genotype	N	%	N	%	*p*
**Arg/Arg**	19	16.10%	4	6.67%	0.0182
**Arg/Gln**	32	27.12%	28	46.67%	>0.05
**Gln/Gln**	67	58.78%	28	46.67%	>0.05

**Table 4:** The frequency of histopathological types of the tumor in the group of patients with lung cancer (n = 118) included in the Arg399Gln polymorphism study of the *XRCC1* gene.

	Adenocarcinoma	Cancer Squamous Cell	Cancer Glandular Squamous	Cancer Large Cell
**Arg/Arg**	12	6	1	0
**%**	63.16%	31.58%	5.26%	0.00%
**Arg/Gln**	19	12	0	1
**%**	59.38%	37.50%	0.00%	3.13%
**Gln/Gln**	33	26	5	3
**%**	49.25%	38.81%	7.46%	4.48%
**all**	64	44	6	4

**Table 5:** Clinical stage of the disease depending on the genotype of the *XRCC1* gene polymorphism studied in the study group (n = 118).

	Tumor Stage I	Tumor Stage II	Tumor Stage III
**Arg/Arg**	11	6	2
**%**	16.92%	14.29%	18.18%
**Arg/Gln**	17	12	3
**%**	26.15%	28.57%	27.27%
**Gln/Gln**	37	24	6
**%**	56.92%	57.14%	54.55%

**Table 6:** Tumor grade rate depending on the genotype of the *XRCC1* gene polymorphism studied in the group of patients with lung cancer (n = 118).

	Tumor Grade G1	Tumor Grade G2	Tumor Grade G1
**Arg/Arg**	1	15	3
**%**	25.00%	15.79%	15.79%
**Gln/Gln**	3	54	10
**%**	75.00%	56.84%	52.63%
**Arg/Gln**	0	26	6
**%**	0.00%	27.37%	31.58%

**Table 7:** Distribution of genotypes depending on the number of pack-years.

	15 Pack/Year N = 17	15–30 Pack/Year N = 36	>30 Pack/Year N = 56
Arg/Arg	5	9	18
%	29.41%	25.00%	32.14%
Arg/Gln	7	12	16
%	41.17%	33.33%	28.57%
Gln/Gln	5	15	22
%	29.41%	41.67%	39.28%

**Table 8:** Results of multivariate analysis of variables associated with the occurrence of cancer in the study group (n = 118).

	Unit/Level	Odds Ratio	95%CI		*p*
**age**	1 year	1.06	1.03	1.10	0.0003
**sex**	men	1.60	0.77	3.34	0.2018
**Arg399Gln *XRCC1***	allele Arg	0.48	0.27	0.85	0.0114
nicotine addiction	current or thrown	3.803	1.42	10.19	0.0079

**Table 9:** Primer sequences and thermal conditions for PCR reactions.

Gene	*XRCC1*
**Polymorphism**	Arg399Gln
**Forward (5′** **→** **3′)**	CAAGTACAGCCAGGTCCTAG
**Reverse (5′** **→** **3′)**	CCTTCCCTCATCTGGAGTAC
**Thermal conditions of amplification**	1. 95 °C—5 min 2. 95 °C—30 s 3. 58 °C—30 s 4. 72 °C—30 s 5. 2 → 3 →4 × 35 cycles 6. 72 °C—3 min
**Product Length (bp)**	248

**Table 10:** A restriction enzyme used in the genotyping of the *XRCC1* gene polymorphism and the length of the fragments obtained after digestion.

Gene	Polymorphism	Enzyme	Fragments After Enzyme Digestion (bp)	Variant Recognized by the Enzyme
***XRCC1***	Arg399Gln	*Bcn*I	159, 89	Arg
			248	Gln
			248, 159, 89	Arg/Gln

## Data Availability

All data and materials, as well as software application, support the published claims and comply with field standards.
